# Real-world advantage and challenge of post-autologous stem cell transplantation MRD negativity in high-risk patients with double-hit multiple myeloma

**DOI:** 10.1186/s12885-024-12077-0

**Published:** 2024-04-02

**Authors:** Yi Tao, Shiwei Jin, Dan Yang, Mengmeng Pan, Wanyan Ouyang, Yuanfang Liu, Yan Wang, Weiping Zhang, Jianqing Mi

**Affiliations:** 1 Shanghai Institute of Hematology, State Key Laboratory of Medical Genomics, Department of Hematology, National Research Center for Translational Medicine at Shanghai, Ruijin Hospital Affiliated to Shanghai Jiao Tong University School of Medicine, 200025 Shanghai, China; 2 Department of Hematology, Lu Daopei Hospital, 200025 Shanghai, China

**Keywords:** Multiple myeloma, Autologous stem-cell transplantation, High-risk cytogenetics, Double hit, Minimal residual disease, Real-world

## Abstract

**Background:**

Autologous stem-cell transplantation (ASCT) remains a beneficial approach for patients with newly diagnosed multiple myeloma (NDMM) in the age of novel therapeutic agents. Nevertheless, limited real-world data is available to establish criteria for identifying high-risk ASCT patients.

**Methods:**

We analyzed outcomes for 168 NDMM patients who underwent ASCT at our center from December 2015 to December 2022. We investigated the impact of the number of high-risk cytogenetics (HRCA), defined as t(4;14), t(14;16), 1q21 gain/amplification, and del(17p), as well as the post-ASCT minimal residual disease (MRD) status as prognostic indicators. We assessed progression-free survival (PFS) and overall survival (OS), and focused on identifying risk factors.

**Results:**

The cohort included 42% of patients (*n* = 71) with 0 HRCA, 42% (*n* = 71) with 1 HRCA, and 16% (*n* = 26) with ≥ 2 HRCA. After a median follow-up of 31 months, the median PFS was 53 months (95% CI, 37–69), and OS was not reached for the entire cohort. Despite similar rates of MRD-negativity post-ASCT, patients with ≥ 2 HRCA, termed “double hit” (DH), had a significantly higher risk of progression/mortality than those with 0 or 1 HRCA. Multivariate analysis highlighted DH (HR 4.103, 95% CI, 2.046–8.231) and MRD positivity post-ASCT (HR 6.557, 95% CI, 3.217–13.366) as adverse prognostic factors for PFS, with DH also linked to inferior OS. As anticipated, DH patients with post-ASCT MRD positivity displayed the poorest prognosis, with a median PFS of 7 months post-ASCT. Meanwhile, DH patients with MRD negativity post-ASCT showed improved prognosis, akin to MRD-negative non-DH patients. It is noteworthy to exercise caution, as DH patients who initially achieved MRD negativity experienced a 41% cumulative loss of that status within one year.

**Conclusions:**

This study strongly advocates integrating DH genetic assessments for eligible ASCT patients and emphasizes the importance of ongoing MRD monitoring, as well as considering MRD-based treatment adaptation for those patients in real-world settings.

**Supplementary Information:**

The online version contains supplementary material available at 10.1186/s12885-024-12077-0.

## Introduction

Over the past two decades, there have been significant therapeutic advancements in treating newly diagnosed multiple myeloma (NDMM) [1, 2]. Despite the introduction of proteasome inhibitors, immunomodulatory drugs, and monoclonal antibodies, as well as the use of high-dose chemotherapy and autologous stem-cell transplantation (ASCT), high-risk NDMM patients still face a poor prognosis [3–5]. With an anticipated increase in options for NDMM, identifying high-risk ASCT patients could inform pre-transplant induction and post-transplant management strategies. The prognosis of MM patients significantly depends on high-risk cytogenetic abnormalities (HRCA) [1, 2]. Clinical trial data consistently indicate adverse outcomes, particularly for patients with concurrent ≥ 2 genetic lesions deemed high-risk, including t(4;14), t(14;16), 1q21 gain/amplification (1q21+), and del(17p), known as “double hit” (DH) [6–8]. The presence of minimal residual disease (MRD) following initial therapy is emerging as a powerful prognostic factor in NDMM [9–11]. However, the significance of combining HRCA number with MRD status as prognostic indicators in real-world ASCT patient population is seldom reported. Our study aims to systematically review the prognostic significance of DH genetics in NDMM patients undergoing standard ASCT at our institution, and to examine the role of post-ASCT MRD status in real-world prognoses.

## Methods

### Patients

We enrolled transplant-eligible patients with NDMM aged 18–65 years with symptomatic, measurable disease defined according to International Myeloma Working Group (IMWG) criteria [12]. We retrospectively reviewed electronic records of 205 patients with NDMM who received ASCT at Ruijin Hospital between December 2015 and December 2022. Cut-off for record review was June 4th, 2023. Cytogenetic risk was centrally assessed by fluorescence in-situ hybridization analysis on CD138-positive sorted cells (200 nuclei analyzed) from bone marrow samples at diagnosis. High risk cytogenetic risk (HRCA) included t(4;14), t(14;16), del(17p), 1q21+ (≥ 3 copies) [13]. The cutoffs for HRCA positivity were 10% for translocations and 20% for copy number aberrations [14]. 168 patients with complete baseline cytogenetic data were included in this analysis and were stratified into three subgroups according to the presence of 0, 1, or ≥ 2 HRCA (double hit).

### Treatment and outcomes

Treatment consisted of up to four phases: induction, ASCT, and four cycles of consolidation, and maintenance. The induction consisted of four 28-day cycles of PAD (bortezomib 1.3 mg/m^2^ on days 1, 4, 8, 11, and liposomal doxorubicin at 30mg/m^2^ (the total dose split over 3 days on cycle 1), plus dexamethasone 40 mg on days 1–4).In 20% of patients, following a suboptimal response (< PR), or in the presence of adverse cytogenetics according to R-ISS stage (i.e., del(17p), t(4;14), or t(14;16)) after two cycles, the induction regimen was intensified with the addition of lenalidomide (10 mg on days 1–21) and/or daratumumab (16 mg/kg on days 1, 8, and 15) of cycles 3–4. 34 patients received treatment intensification: 23% of patients with 0 HRCA, 8% of 1 HRCA, and 46% of DH patients (Table 1). Upon completion of induction, patients proceeded with collection of autologous hematopoietic cells using CE regimen (cyclophosphamide 30 mg/kg/d×2d, VP-16 5 mg/kg/d×2d) followed by granulocyte colony-stimulating factor (G-CSF) with or without plerixafor as mobilization. Patients then underwent ASCT conditioned with high dose melphalan (140–200 mg/m^2^). After subsequent response assessment (within 100 days post-ASCT), those with response less than CR received four cycles of VCD (bortezomib 1.3 mg/m^2^ on days 1, 4, 8 and 11, cyclophosphamide 750mg/m^2^ intravenously on day 1, and dexamethasone 40 mg on days 1, 8, 15 and 22 on a 28-day cycle) consolidation, followed by maintenance. Post-transplant maintenance was used with lenalidomide 10 mg days 1–21 every 28 days and/or bortezomib 1.3 mg/m^2^ once every 2 weeks for 2 years.

**Table 1:** Clinical characteristics

Characteristic No. (%)	0 h-CA *N* = 71 (42%)	1 h-CA *N* = 71 (42%)	≥ 2 h-CA *N* = 26 (16%)	Total *N* = 168
Age, years				
Median (range)	55 (26–67)	55 (26–67)	55 (36–65)	55 (26–67)
Age > 60, No. (%)	15 (21%)	23 (32%)	9 (35%)	47 (28%)
Sex, No. (%)				
Male	35 (49%)	36 (51%)	12 (46%)	83(49%)
Female	36 (51%)	35 (49%)	14 (54%)	85 (51%)
ISS stage, No. (%)				
Stage1	36 (51%)	24 (34%)	6 (23%)	66(39%)
Stage2	20 (28%)	25 (35%)	14 (54%)	59 (35%)
Stage 3	15 (21%)	22 (31%)	6 (23%)	43 (26%)
R-ISS stage, No. (%)				
Stage1	26 (37%)	10 (14%)	2 (8%)	38(23%)
Stage2	40 (56%)	51 (72%)	18 (69%)	109 (65%)
Stage 3	5 (7%)	10 (14%)	6 (23%)	21 (13%)
Induction regimen, No. (%)				
PI-based	55 (77%)	65 (92%)	14 (54%)	134 (80%)
PI and IMiD combination	12 (17%)	3 (4%)	9 (35%)	24 (14%)
PI, IMiD and CD38 Ab combination	4 (6%)	3 (4%)	3 (11%)	10 (6%)
Response before ASCT, No. (%)				
≥VGPR	49 (69%)	54 (76%)	22 (85%)	125 (74%)
<VGPR	22 (31%)	17 (24%)	4 (15%)	43 (26%)
Response after ASCT, No. (%)				
MRD negative	39 (55%)	37 (52%)	14 (54%)	90 (54%)
MRD positive	32 (45%)	34 (48%)	12 (46%)	78 (46%)
Maintenance regimen, No. (%)				
Bortezomib	54 (76%)	57 (80%)	14 (54%)	125 (74%)
Lenalidomide	9 (13%)	8 (11%)	3 (12%)	20 (12%)
Bortezomib + Lenalidomide	6 (9%)	5 (7%)	8 (31%)	19 (11%)
High risk cytogenetics, No. (%)				
Gain or amplification of 1q21	NA	57 (80%)	24 (92%)	81 (48%)
del(17p)	NA	9 (13%)	8 (31%)	17 (10%)
t(4;14)	NA	5 (7%)	20 (77%)	25 (15%)
t(14;16)	NA	0 (0%)	1 (4%)	1 (1%)
Other clinical characteristics, No. (%)				
Elevated LDH	17 (24%)	18 (25%)	13 (50%)	48 (29%)
Renal dysfunction (Ccr < 60 ml/min)	12 (17%)	11 (16%)	4 (15%)	27 (16%)
Hypercalcemia	7 (10%)	5 (7%)	2 (8%)	14 (8%)
Anemia	20 (28%)	31 (44%)	10 (39%)	61(36%)
Osteolytic lesions	59 (83%)	59 (83%)	22 (85%)	140 (83%)
Extramedullary disease (bone-related)	17 (24%)	11 (16%)	3 (12%)	31 (19%)
Extramedullary disease (extraosseous)	5 (7%)	0 (0%)	2 (8%)	7 (4%)

Progression-free survival (PFS) was defined as time from the initialization of therapy, or from stem cell re-infusion (post-ASCT) to progression as per IMWG criteria [12] or death by any cause. Overall survival (OS) was defined as time from the initialization of therapy, or from stem cell re-infusion (post-ASCT) to death by any cause. Patients who did not progress, died, were lost to follow-up were censored at the time of the last disease assessment. In this retrospective analysis, we investigated the effect of HRCA numbers (0, 1, or ≥ 2) on PFS and OS in the overall patient population. We also assessed the PFS and OS from the date of stem cell re-infusion in patients stratified according to the presence of DH (≥ 2 HRCA) at diagnosis and MRD status after ASCT. Depth of response and relapse criteria were defined as per the IMWG criteria [15].

The evaluation of minimal residual disease (MRD) status was performed using a one-tube 10-color assay by FACSCanto flow cytometer (Becton Dickinson, San Jose, CA, USA) with the antibody combination including cytoplasmic-kappa-FITC/cytoplasmic-PE/CD117-PE-CY5.5/CD19-PC7/CD138-APC/CD56-APC-A700/CD45-APC-H7/CD81-PB/CD27-BV605/CD38-BV510. The limit of detection (LOD) achieved by flow cytometric approach was calculated in each sample according to the following formula: (20/number of viable nucleated cells) × 100 [16]. At least one million cellular events for each sample were acquired to reach a theoretical LOD of 2.0 × 10^− 5^. Data were analyzed using the Kaluza Analysis Software to determine the percentage of phenotypically aberrant clonal plasma cells (PCs) in total nucleated cells. MRD positivity was defined as the percentage of phenotypically aberrant clonal PCs equal to or greater than the LOD, otherwise, MRD negativity was considered. MRD negativity refer to CR-based undetectable MRD as per IMWG criteria [15]. Evaluation of MRD was done after 4 cycles of induction, before ASCT, within 100 days post-ASCT, and thereafter during follow-up in all patients if possible. For patients reaching MRD negative post ASCT, we monitored MRD status in the bone marrow every 6 months for the first year and yearly thereafter.

### Statistics

Time-to-event end points were estimated using the Kaplan-Meier method and compared using pairwise log-rank test. Cox proportional hazard regression was used to estimate univariate and multivariate hazard ratios (HR) and 95% confidence intervals (CI). Multivariable logistic regression was used to assess factors associated with post-ASCT MRD status. To assess MRD resurgence or progression across different risk strata, we employed the cumulative incidence function, accounting for death without progression as a competing event. The risks of MRD resurgence or progression were compared using the Gray test. Point estimates for binary endpoints are reported with 95% CI using the Clopper-Pearson exact method, which is based on a binomial distribution. All statistical analyses were conducted using the SPSS software (version 22.0) and R packages survival & survminer in R/Bioconductor (version 3.6.1), with significance defined as *p* < 0.05.

## Results

### Clinical and genetic characteristics

From December 2015 to December 2022, our center administered ASCT to 205 patients with NDMM. Among these, 168 patients (82%) with complete cytogenetic data were included in the analysis. Table 1 provides a comprehensive overview of the clinical and genetic attributes of these patients. Notably, 48% of patients exhibited 1q21+, 10% with del(17p), 15% with t(4;14), and 1% with t(14;16). Regarding the number of HRCA, there were 42%, 42%, and 16% of patients with 0, 1, and ≥ 2 HRCA, respectively. The median age was 55 years (range, 26–67 years), with 28% being over 60 years of age. Furthermore, 49% were male, 26% presented with ISS stageIII, and 13% with R-ISS stageIII. Clinically, 29% of patients presented with an increased serum lactate dehydrogenase (LDH), renal impairment (GFR < 60 mL/min) was noted in 16%, hypercalcemia in 8%, anemia in 36%, and osteolytic lesions in 83% of individuals. Furthermore, 19% were in the bone-related extramedullary group and 4% in the extraosseous extramedullary group. In terms of induction regimen, the majority (80%) were treated with a proteasome inhibitor (PI)-based regimen, 14% with a PI/immunomodulatory drug (IMiD) combination regimen, and 6% with a PI/IMiD/CD38-Antibody (Ab) combination. For maintenance therapy, 74% were on bortezomib; 12% on lenalidomide; and another 11% on a combination of bortezomib and lenalidomide. Prior to ASCT, 74% of patients achieved at least a very good partial response (≥ VGPR), and post-ASCT, 92% attained ≥ VGPR, with 54% achieving MRD-negative CR (Table 1). Enhanced response depths were observed sequentially after induction, following ASCT, during consolidation, and/or throughout the maintenance stage for the respective high-risk groups (Fig. S1).

The concurrence of HRCA in the subset of 26 DH (≥ 2 HRCA) patients is depicted (Fig. S2). Notably, 65% of DH patients (*n* = 17) exhibited a concurrence of 1q21 + and t(4;14), 19% (*n* = 5) had a concurrence of 1q21 + and del(17p), 8% (*n* = 2) with a concurrence of t(4;14) and del(17p), and 4% (*n* = 1) with a concurrence of 1q21 + and t(14;16). Among these, 4% (*n* = 1) demonstrated a triple hit (3 HRCA), showing a co-existence of 1q21+, del(17p), and t(4;14).

### Genetic contributors to outcome of patients receiving ASCT

**Fig. 1:**
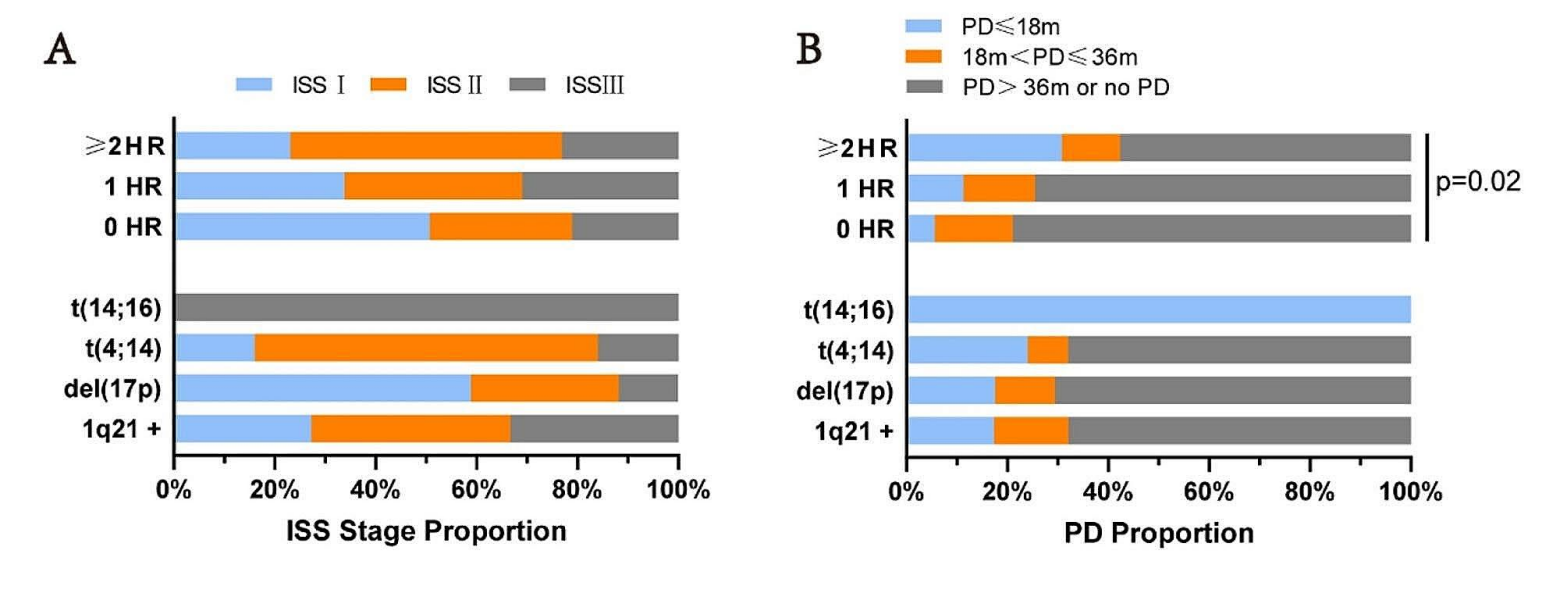
The association of individual HRCA or the number of HRCA with clinical risk groups. (**A**) The distribution of individual HRCA or the number of HRCA by ISS sorted by the proportion of patients; (**B**) The contribution of individual HRCA or the number of HRCA to relapse sorted by the proportion of patients, with a breakdown of PFS over ≤ 18 months/ 18–36 months/>36 months, or no progression. HR, high risk; HRCA, high-risk cytogenetics; PD, progressive disease

To assess the contribution of individual HRCA or their number to the risk status, we plotted these factors against markers of risk such as ISS and time to relapse. The distribution of specific HRCA or their number demonstrated no dependency on ISS stage (Fig. 1A). This suggests a lack of predictability of ISS stage based on specific HRCA or their quantity, and vice versa. The distribution of each HRCA and their number in relation to time to relapse, along with the percentage breakdown of relapse timing, is depicted. Notably, 31% of patients with ≥ 2 HRCA experienced progression within 18 months, significantly higher than those with 0 HRCA (5.6%, *p =* 0.001) or 1 HRCA (11.2%, *p =* 0.022) (Fig. 1B). The disparities in early relapse (≤ 18 months) rates based on the number of HRCA suggest a rationale for incorporating DH cytogenetics into predictive models for unfavorable patient outcomes.

### Prognosis for NDMM patients stratified by the number of HRCA treated with ASCT

**Fig. 2:**
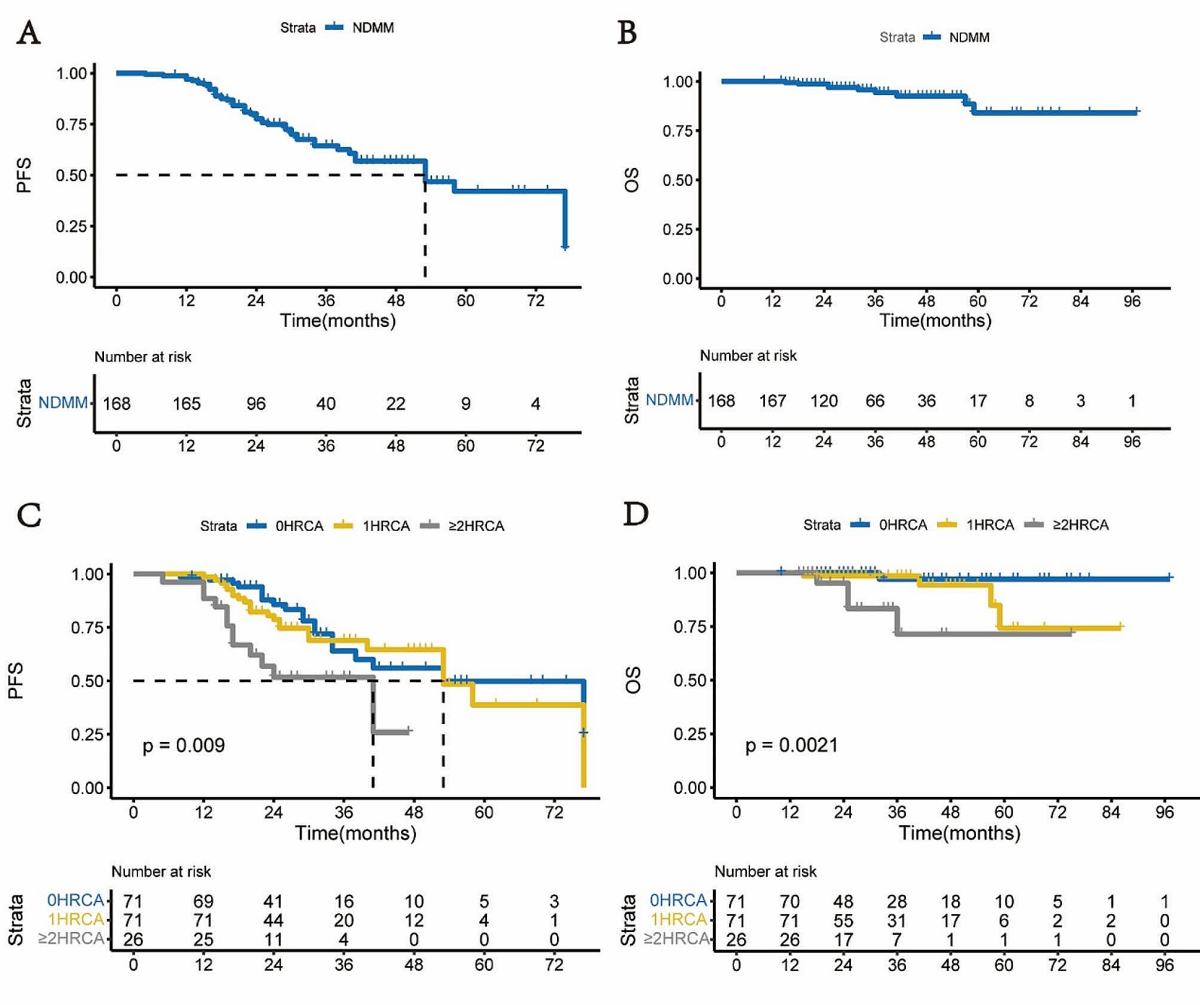
Kaplan-Meier plots for PFS and OS for NDMM patients treated with standard of care ASCT. (**A**, **B**) PFS (**A**) and OS (**B**) for the entire cohort of 168 patients; (**C**, **D**) PFS (**C**) and OS (**D**) stratified by the number of HRCA (0 vs. 1 vs. ≥2 HRCA). HRCA, high-risk cytogenetics; PFS, progression free survival; OS, overall survival

With a median follow-up of 31 months, the median PFS of overall ASCT patients was 53 months (95% CI, 37–69) (Fig. 2A and B). Patients with DH MM had a significantly shorter median PFS (41 months, 95% CI, 18–64) compared to those with 0 HRCA (53 months, 95% CI, 31–75, *p =* 0.005) and 1 HRCA (53 months, 95% CI, 38–68, *p =* 0.013), who displayed similar outcomes (*p =* 0.461) (Fig. 2C). The median OS was not reached overall, with an estimated 3-year OS rate of 71% for DH MM, significantly inferior to those with 0 HRCA (3y-OS 97%, *p =* 0.003) and 1 HRCA (3y-OS 99%, *p =* 0.020), who also exhibited comparable OS rates (*p =* 0.134) (Fig. 2D). Regarding the impact of individual HRCA, patients with single 1q21+ (*n* = 57) exhibited similar PFS (HR 1.294, 95% CI, 0.684–2.448, *p =* 0.428) and OS (HR 3.098, 95% CI, 0.280-34.317, *p =* 0.357) to those with 0 HRCA (*n* = 71) (Fig. S3). Patients with single del(17p) (*n* = 9) also had comparable PFS (HR 1.218, 95% CI, 0.358–4.143, *p =* 0.752) and OS (HR 5.646, 95% CI, 0.349–91.232, *p =* 0.223) (Fig. S4). The subgroup with single t(4;14) (*n* = 5) showed no significant difference in PFS (HR 0.800, 95% CI, 0.106–6.013, *p =* 0.828), but inferior OS (HR 32.860, 95% CI, 1.678-643.387, *p =* 0.021) (Fig. S5) compared to those with 0 HRCA.

### Univariate and multivariate cox regression

**Fig. 3:**
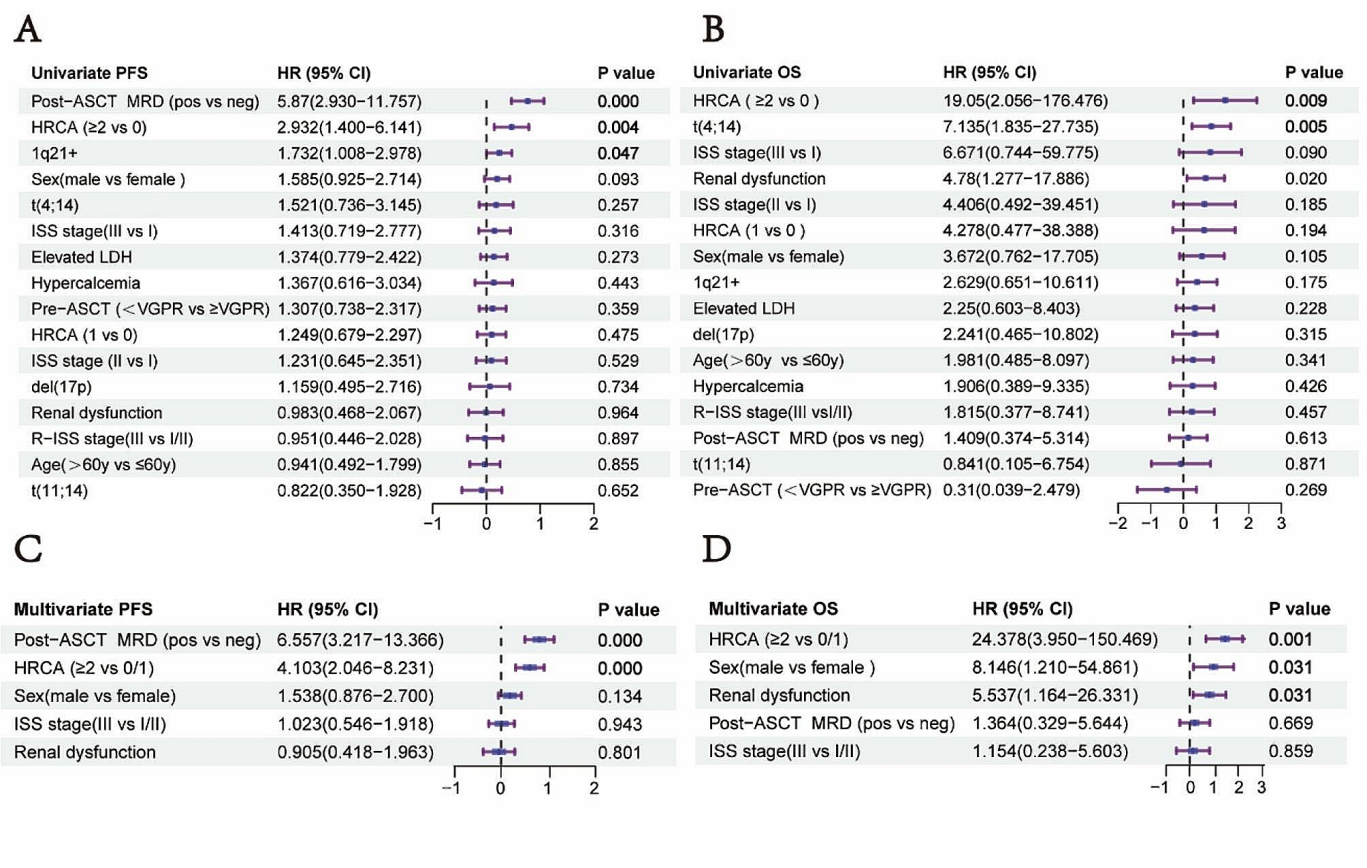
Univariate and multivariate analyses. (**A**, **B**) Associations of clinical factors and the number of HRCA with PFS (**A**) and OS (**B**) in univariate analyses; (**C**, **D**) Associations of clinical factors and the number of HRCA with PFS (**C**) and OS (**D**) in multivariate analyses. HRCA, high-risk cytogenetics; HR, hazard ratio; CI, confidence interval; PFS, progression free survival; OS, overall survival; HR was transformed into log_10_(HR) in the Figure

To ascertain contributing factors to an unfavorable prognosis, we conducted an analysis in 168 patients with complete PFS, OS, and clinical characteristics. Covariates encompassed age, sex, ISS, R-ISS, individual HRCA and their number (t(14;16) was excluded for only 1 patient), pre- and post-ASCT response, elevated LDH, and hypercalcemia. Univariate analyses revealed that adverse PFS was associated with DH cytogenetics (HR 2.932, 95% CI, 1.400-6.141, *p =* 0.004), post-ASCT MRD positivity (HR 5.870, 95% CI, 2.930-11.757, *p =* 0.000), and 1q21+ (HR 1.732, 95% CI, 1.008–2.978, *p =* 0.047) (Fig. 3A), while DH cytogenetics (HR 19.050, 95% CI, 2.056-176.476, *p =* 0.009), and t(4;14) (HR 7.135, 95% CI, 1.835–27.735, *p =* 0.005) were significantly associated with reduced OS (Fig. 3B). In multivariate analyses, which incorporated covariates selected from univariate results (*p* < 0.10), both DH cytogenetics (HR 4.103, 95% CI, 2.046–8.231, *p =* 0.000) and post-ASCT MRD positivity (HR 6.557, 95% CI, 3.217–13.366, *p =* 0.000) emerged as independent predictors of unfavorable PFS (Fig. 3C). Moreover, reduced OS was independently associated with DH cytogenetics (HR 24.378, 95% CI, 3.950-150.469, *p =* 0.001), male gender (HR 8.146, 95% CI, 1.210-54.861, *p =* 0.031), and renal dysfunction (HR 5.537, 95% CI, 1.164–26.331, *p =* 0.031) (Fig. 3D).

### Response to therapy in DH patients

**Fig. 4:**
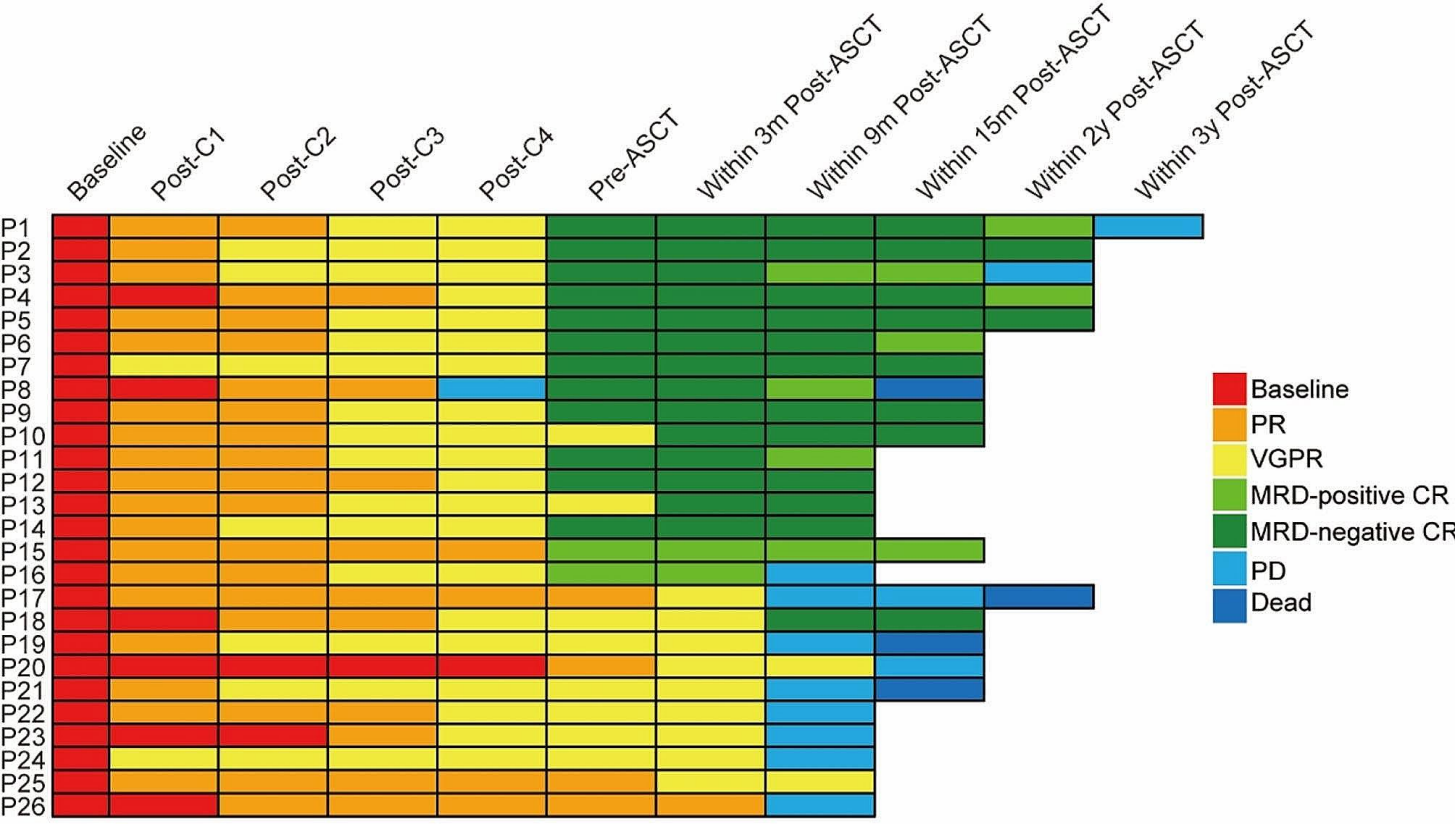
Response to therapy, by number of cycles and follow-up in the subset of 26 DH (≥ 2HRCA) patients. P, patient; C, cycle; m, months; y, years; PR, partial remission; VGPR, very good partial remission; CR, complete remission; PD, progressive disease

Among the 26 DH patients receiving induction therapy, 54% received PI-based regimens, 35% were treated with PI/IMid, and 11% had a PI/IMid/CD38 Ab combination therapy. As illustrated in Fig. 4, individual patients exhibited a deepening clinical response over time. The overall response rate (≥ PR) following two induction cycles was 92% (24 of 26 patients), and the rate of achieving ≥ VGPR before ASCT was 85% (22 of 26 patients), which increased to 96% (25 of 26 patients) post-ASCT. Similar to patients with 0 (55%) or 1 HRCA (52%), 54% of the DH patients (14 of 26) exhibited MRD-negative CR in the bone marrow within 3 months post-ASCT, being 50% (7 of 14), 56% (5 of 9) and 67% (2 of 3) for those treated with PI, PI/IMid, and PI/IMid/CD38 Ab-based induction, respectively. Notably, of the 10 DH patients who achieved less than CR after ASCT, 80% experienced subsequent disease progression, with 70% progressing within 9 months post-ASCT.

Among 26 DH patients, four patients (patients 8,17,19, and 21) succumbed after ASCT. Three patients (P17, P19, and P21), each carrying both 1q21 + and t(4;14), attained VGPR post-ASCT but experienced rapid progression (7,7 and 4 months post-ASCT, respectively) during post-ASCT consolidation therapy. Ultimately, all three patients developed extramedullary chest involvement and significant pleural effusion before they succumbed to disease progression and multi-organ failure. One patient (P8), who possessed 1q21 + and del(17p), developed central nervous system (CNS) involvement after four cycles of PAD induction. This patient achieved MRD negativity in both the bone marrow and CNS following two cycles of temozolomide and VRD (bortezomib, lenalidomide, dexamethasone) combination therapy, and then underwent ASCT. Although MRD negativity was maintained post-ASCT, the patient progressed to plasma cell leukemia during PI/IMid maintenance therapy 10 months post-ASCT and died 4 months thereafter.

### Prognosis for NDMM patients according to the presence of DH HRCA and post-ASCT MRD status

**Fig. 5:**
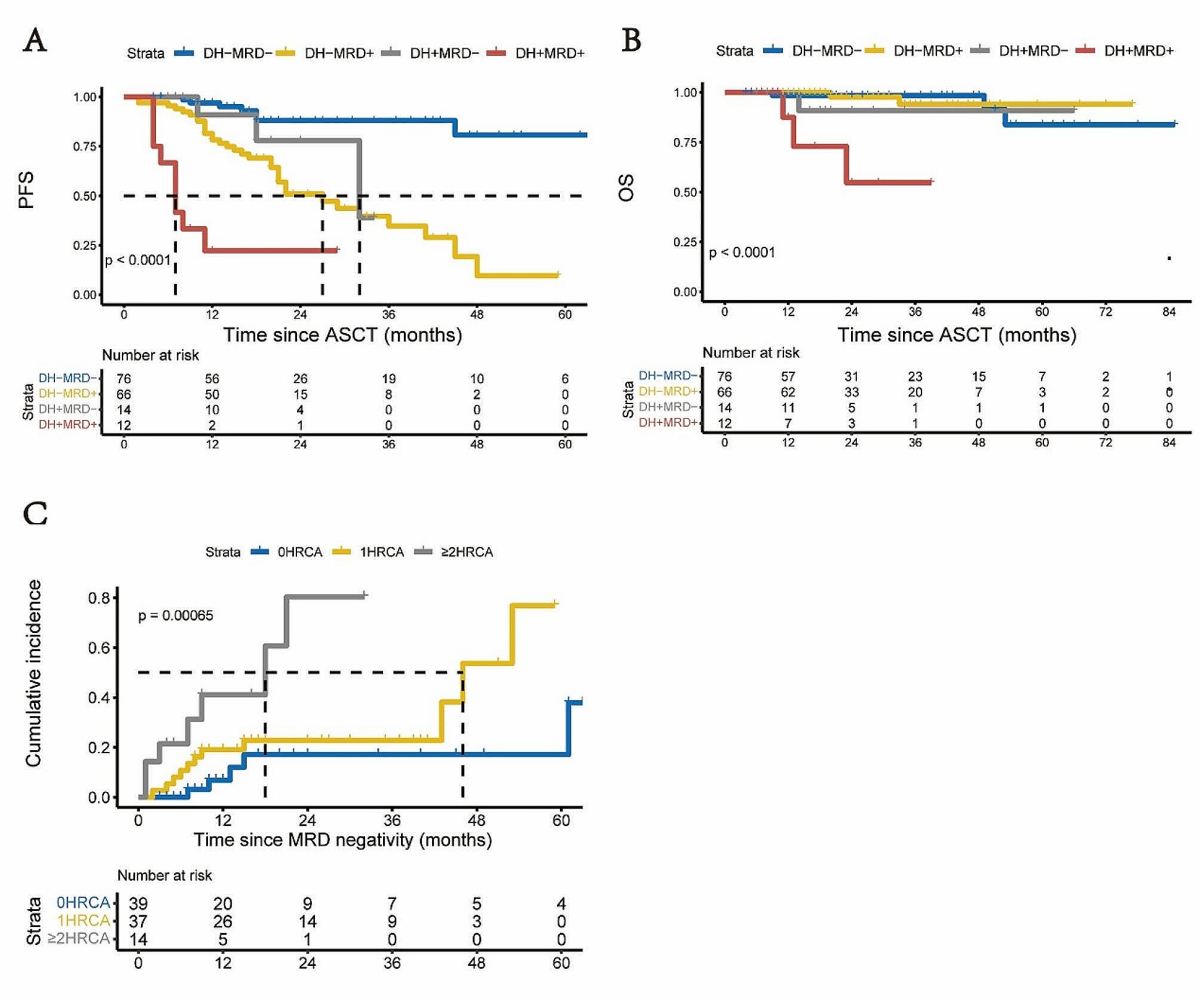
Prognosis of patients according to the presence of DH HRCA and post-ASCT MRD status. (**A**, **B**) Probability of PFS (**A**) and OS (**B**) adjusted for the presence of DH HRCA and post-ASCT MRD status; (**C**) Cumulative incidence of progression or MRD resurgence for patients stratified by the number of HRCA after achieving post-ASCT MRD negativity. DH, double hit; MRD, minimal residual disease; HRCA, high-risk cytogenetics; PFS, progression free survival; OS, overall survival

Upon further stratifying patients into four groups based on the presence of DH HRCA and post-ASCT MRD status, findings revealed that both non-DH and DH patients experienced enhanced PFS upon achieving MRD negativity post-ASCT, compared to their MRD-positive counterparts (MRD- vs. MRD + in non-DH, *p =* 0.000; MRD- vs. MRD + in DH, *p =* 0.002). Importantly, DH patients who achieved MRD negativity (DH + MRD-) exhibited PFS improvement from the time of transplantation, similar to non-DH patients who achieved MRD negativity (DH-MRD-) (*p =* 0.114). While there was no statistically significant difference in PFS between DH + MRD- and DH-MRD + patients (*p =* 0.305), a trend towards extended survival was observed in DH + MRD- (2-year PFS post-ASCT of 79.6% vs. 50.9%). As anticipated, patients with DH + MRD + displayed the poorest prognosis, with a median PFS of 7 months post-ASCT (Fig. 5A). In terms of OS, patients with DH + MRD- demonstrated comparable OS to those with DH-MRD- (*p =* 0.391), whereas those with DH + MRD + had a dismal OS compared to DH-MRD- patients (*p* < 0.001), with a 2-year OS post-ASCT of 55% (Fig. 5B).

In total, out of the 90 patients achieving MRD negativity post-ASCT, 39, 37, and 14 patients had 0, 1, and ≥ 2 HRCA, respectively. The median follow-up after MRD negativity was 17 months. The 1-year cumulative incidence of progression or MRD resurgence was 41% for patients with ≥ 2 HRCA (HR 8.758, 95% CI, 2.451–31.299, *p =* 0.001), 19% for those with 1 HRCA (HR 2.824, 95% CI, 0.893–8.924, *p =* 0.077), and 7% for those with 0 HRCA (Fig. 5C).

## Discussions

Our definition of HRCA encompasses 1q21 + along with t(4;14), t(14;16) and del(17p), in line with established standards in the field [7, 17, 18]. In our cohort, 16% patients exhibited DH genetics (≥ 2 HRCA), consistent with the 20% reported in the Master trial [8]. Similar to a recent report from MD Anderson, the most common combinations of HRCA in DH cases were [1q+, t(4;14)] and [1q+, del(17p)] [19]. Our findings reveal that DH patients had a higher risk of progression or death than patients with 0 or 1 HRCA despite achieving similar rates of MRD-negativity post-ASCT, thereby representing an unmet medical need. The increased risk in DH patients could be attributed to both worse outcomes in those who remain post-ASCT MRD-positive and a higher rate of MRD negativity loss, even in those who initially reached post-ASCT MRD negativity. Importantly, in multivariate analysis, the presence of ≥ 2 HRCA has emerged as an independent prognostic factor, thereby establishing a robust association between DH genetics and poor outcomes in the context of ASCT. We also observed that a bortezomib-based regimen combined with ASCT as standard treatment for NDMM resulted in comparable PFS and OS rates in patients with 0 or 1 HRCA, effectively neutralizing the elevated risk of progression or death associated with a single HRCA as a whole. Although our data suggest that 1q21 + predicts inferior PFS and that t(4;14) is associated with worse OS in univariate analysis, it is noteworthy that 30% of 1q21 + cases and 80% of t(4;14) cases fell within the DH category. Despite the widely acknowledged adverse effect of 1q21+ [20–22], the prognostic significance of a singular occurrence in the context of ASCT is still undefined. Our analysis did not reveal a negative impact of single 1q21 + on prognosis compared to those with 0 HRCA among ASCT patients. Given the small number of patients (*n* = 5), the detrimental effect of single t(4;14) compared to 0 HRCA on OS requires further investigation. Recent results from the FORTE trial, which involved carfilzomib-based triplet regimens with ASCT as initial treatment, validated a worse PFS in patients with ≥ 2 HRCA [7].

Our analysis shows that stratification by the number of HRCA (DH vs. non-DH) and MRD status (MRD-negative vs. MRD-positive) enhances risk classification further. This stratification uncovers a subgroup with excellent and comparable PFS and OS outcomes, notably those who attain MRD negativity post-ASCT, irrespective of DH status, and another with an absolutely inferior prognosis - DH-positive and post-ASCT MRD-positive (DH + MRD+) patients. This observation indicates that achieving MRD negativity after ASCT could alleviate or even neutralize DH risk factors established at diagnosis. Supporting this notion, a recent meta-analysis covering studies from January 1990 to January 2016 demonstrated that MRD negativity was found to confer an approximate 50% relative reduction in both progression and mortality risk [23]. These findings, along with our analysis showing that disease stage and cytogenetic risk profiles had no significant impact on post-ASCT MRD negativity rates (Fig. S6), underscore the necessity of discussing prognosis with patients not only at diagnosis but also throughout treatment phases, as their prognosis could be substantially altered by MRD negative status. Given the dismal prognosis for MRD-positive DH patients, enhancing induction regimens is essential. In quadruplet regimens combined with ASCT, exemplified by CASSIOPEIA [24] and GRIFFIN [25] trials, the incorporation of CD38 Ab into induction protocols greatly increased MRD negativity post-ASCT, from 44% with VTd to 64% with D-VTd, or from 20% with VRd to 51% with D-VRd. Furthermore, the MASTER study revealed that 79% of DH patients achieved MRD negativity by NGS (cutoff 10^− 5^) following Dara-KRd induction, ASCT, and Dara-KRd consolidation [8]. Considering that some DH patients remain MRD-positive despite intensified induction and ASCT, pioneering treatments like chimeric antigen receptor T-cells (CART) and bispecific T-cell engagers moving to frontline, may be justified. As shown in our earlier reports, B-cell maturation antigen–targeting (BCMA)-CART is promising, offering deep remission (72.9% MRD-negative CR, cutoff 10^− 5^) and a favorable long-term safety profile in relapsed/refractory MM [26–28].

Nevertheless, achieving MRD negativity post-ASCT in DH patients has been shown to be insufficient. Our findings suggest that it is challenging for DH patients to sustain MRD negativity. One potential explanation for this may be the limited sensitivity of flow cytometry sensitivity (2 × 10^− 5^) employed in our study. However, the MASTER trial, implementing a higher sensitivity threshold of < 10^− 5^ by NGS, also reported a higher risk of losing MRD negativity in MRD-negative DH patients. Similarly, the FORTE trial [7], used the same MRD sensitivity as MASTER, revealing a significantly lower rate of 1-year sustained MRD negativity in DH patients. Whereas, those DH who maintained 1-year sustained MRD negativity showed a 4-year PFS comparable to patients with 0 or 1 HRCA [7]. This underscores the critical importance of sustained MRD negativity as a treatment goal for DH patients. With a longer median follow-up in our study, it is plausible that DH + MRD- patients might exhibit a significantly distinct survival curve from those DH-MRD-, owing to a lower rate of sustained MRD negativity. As a result, we strongly advise against treatment deintensification for DH patients solely based on achieving MRD negativity, particularly outside of clinical trial settings. Furthermore, post-transplant changes in MRD status across varying numbers of HRCA highlight the necessity for serial MRD assessments in initially MRD-negative patients to better assess progression risk. While the introduction of CD38 Ab-based quadruplet regimens is likely to enhance the initial response rates in DH patients, maintaining sustained MRD negativity requires tailored post-ASCT intensified treatment. Analyses from the MASTER, FORTE, and UK OPTIMUM/MUKnine trials advocate risk-adapted post-ASCT consolidation for this patient cohort [8, 29, 30]. Moreover, adding carfilzomib to lenalidomide maintenance extends PFS beyond lenalidomide alone, across all cytogenetic risk groups in the FORTE [7], further supporting intensified maintenance for DH patients. This includes using a doublet of a proteasome inhibitor and an immunomodulatory drug or even more potent combinations.

A limitation of our study is that patients received PAD induction therapies, with the addition of lenalidomide and/or CD38 Ab later. The initial choice of PAD rather than VRD as the standard of care was pragmatic, based on insurance coverage. Despite different induction regimens, our analysis showed similar and high rates of post-ASCT MRD negativity across various risk strata. Furthermore, the ISKIA trial presented at this year’s ASH indicates that the addition of isatuximab to the KRd regimen increased the rate of post-consolidation MRD negativity at the 10^− 5^ level to a small extent (77% for IsaKRd vs. 67% for KRd; *p* = 0.049), but demonstrated a more pronounced difference at the 10^− 6^ threshold (67% vs. 48%; *p* < 0.001), better distinguishing between the two treatment groups. This improvement was consistent among DH patients (10^− 5^: 77% vs. 53%; 10^− 6^: 77% vs. 27%). These findings suggest that deeper MRD thresholds are crucial to accurately identify MRD negativity in patients including in those with DH [31].

In summary, our findings strongly advocate the incorporation of DH genetics assessment for all eligible ASCT patients in real-world scenarios. The data suggest DH patients might initially benefit from achieving post-ASCT MRD negativity via tailored pre-transplant induction, followed by maintaining this negativity through post-transplant consolidation and intensive maintenance. For DH patients, even after initial post-ASCT MRD negativity is achieved, continuous MRD monitoring remains essential.

### Electronic supplementary material

Below is the link to the electronic supplementary material.

Supplementary Material 1

## Data Availability

The data generated in this study are available within the article and its supplementary data files.
