# Supplementary material for: Real-world advantage and challenge of post-autologous stem cell transplantation MRD negativity in high-risk patients with double-hit multiple myeloma
Source: BMC Cancer. 2024 Apr 2;24:406. doi: 10.1186/s12885-024-12077-0 (PMC10985970; doi:10.1186/s12885-024-12077-0)
Supplement: Supplementary file 1 — Supplementary Material 1 [file 12885_2024_12077_MOESM1_ESM.docx]

**Supplementary data**


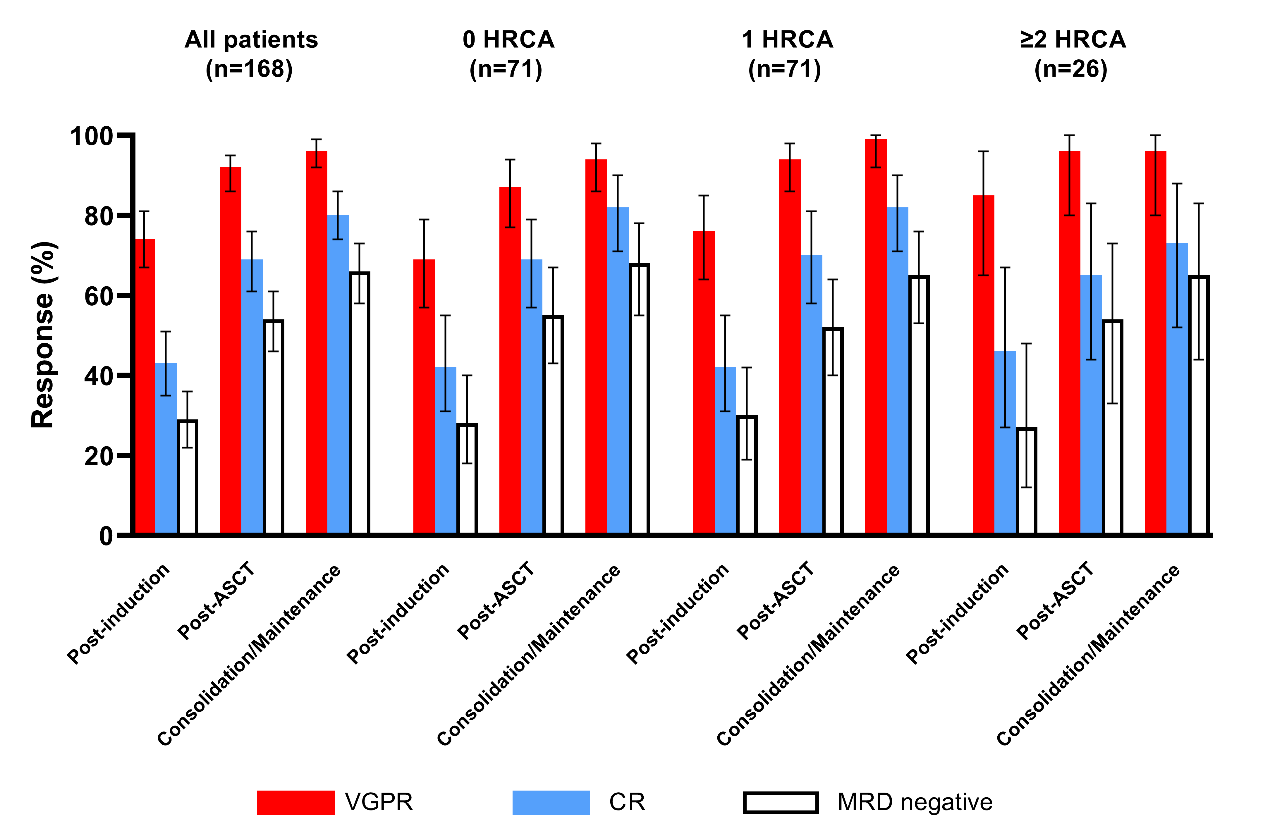


**Fig. S1.** Enhanced response depths observed sequentially pos-induction, post-ASCT, during consolidation, and/or throughout the maintenance for patients with 0,1, ≥2 HRCA。


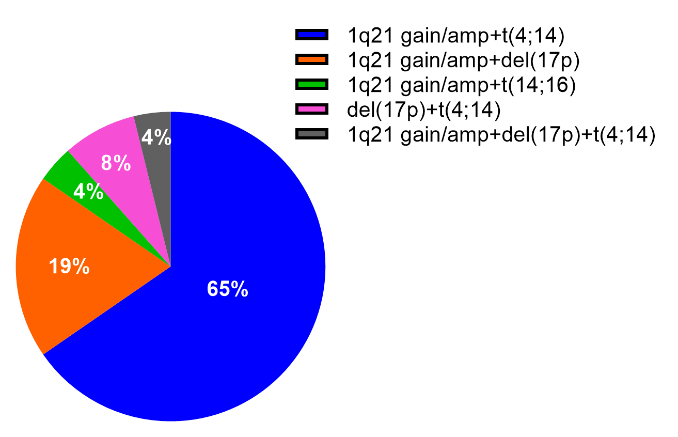


**Fig. S2 The distribution of concurrence of HRCA in the subset of 26 DH (≥2HRCA) patients.**


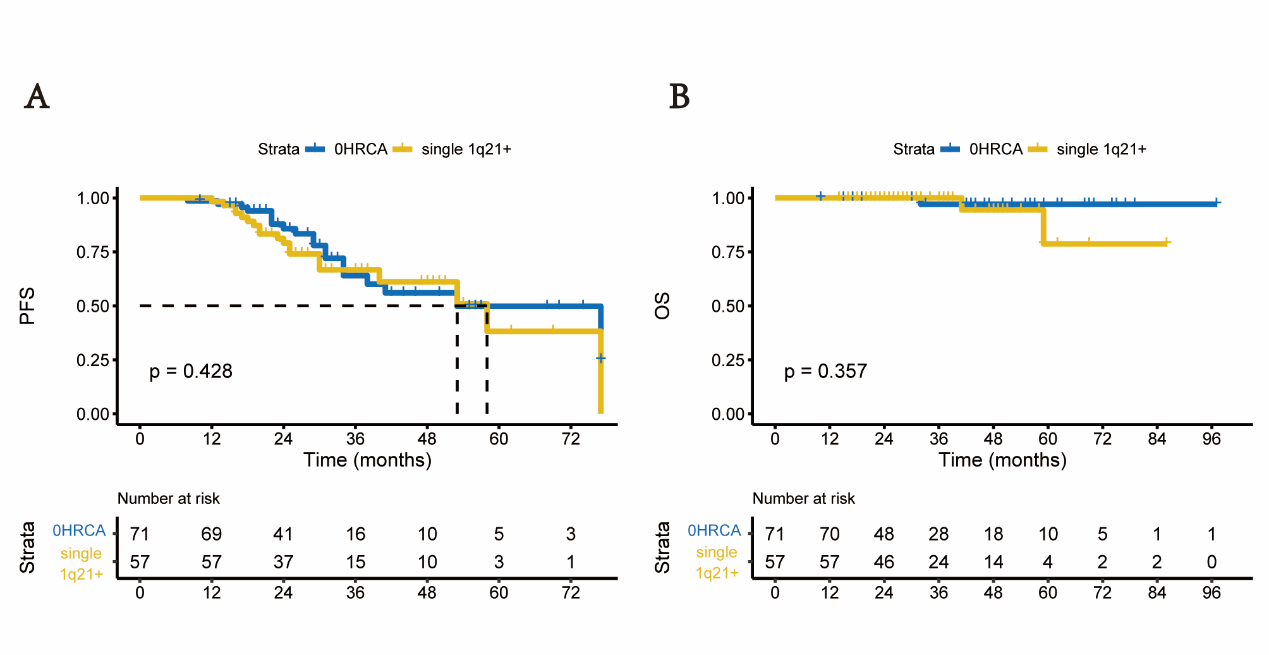


**Fig. S3 Prognosis of patients with single 1q21+ compared to those with 0HRCA.**

A, B. Kaplan-Meier plots for PFS (A) and OS (B) for NDMM patients with single 1q21+ compared to those with 0HRCA in the setting of ASCT.

**
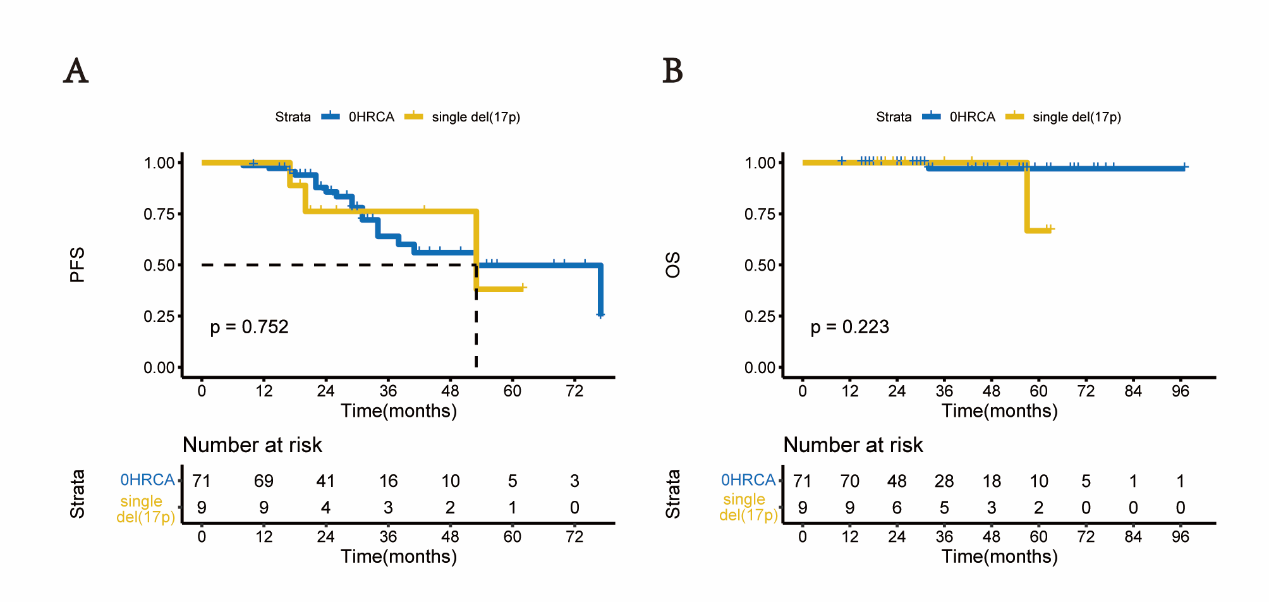
**

**Fig. S4 Prognosis of patients with single del(17p) compared to those with 0HRCA.**

A, B. Kaplan-Meier plots for PFS (A) and OS (B) for NDMM patients with single del(17p) compared to those with 0HRCA in the setting of ASCT.

**
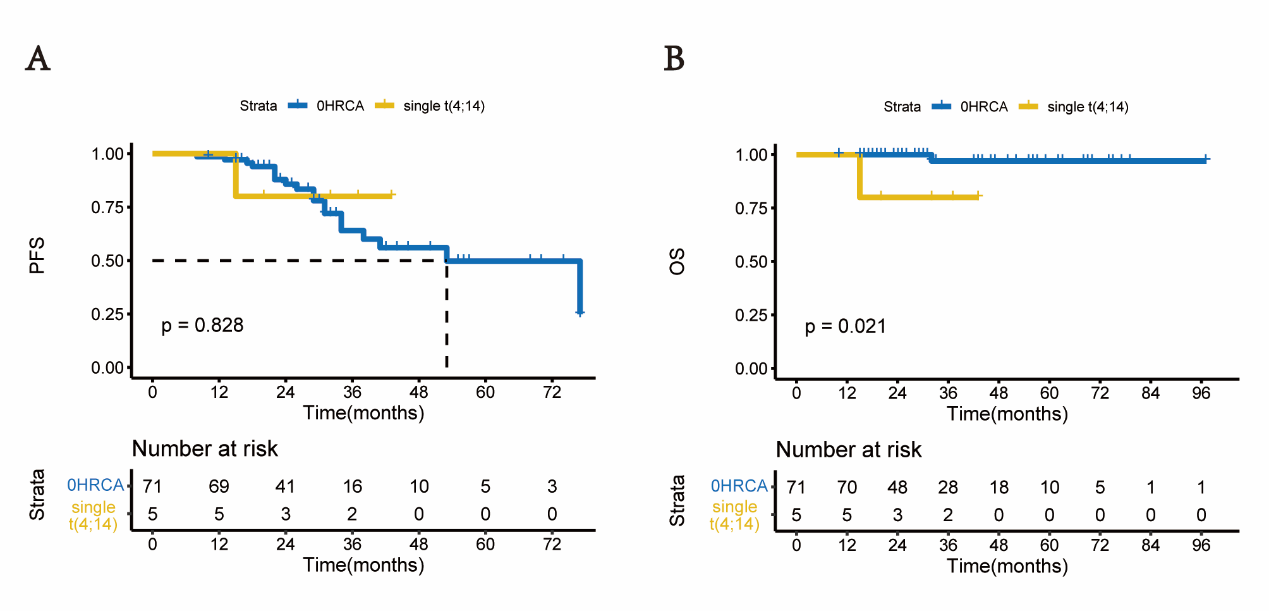
**

**Fig. S5 Prognosis of patients with single t(4;14) compared to those with 0HRCA.**

A, B. Kaplan-Meier plots for PFS (A) and OS (B) for NDMM patients with single t(4;14) compared to those with 0HRCA in the setting of ASCT.


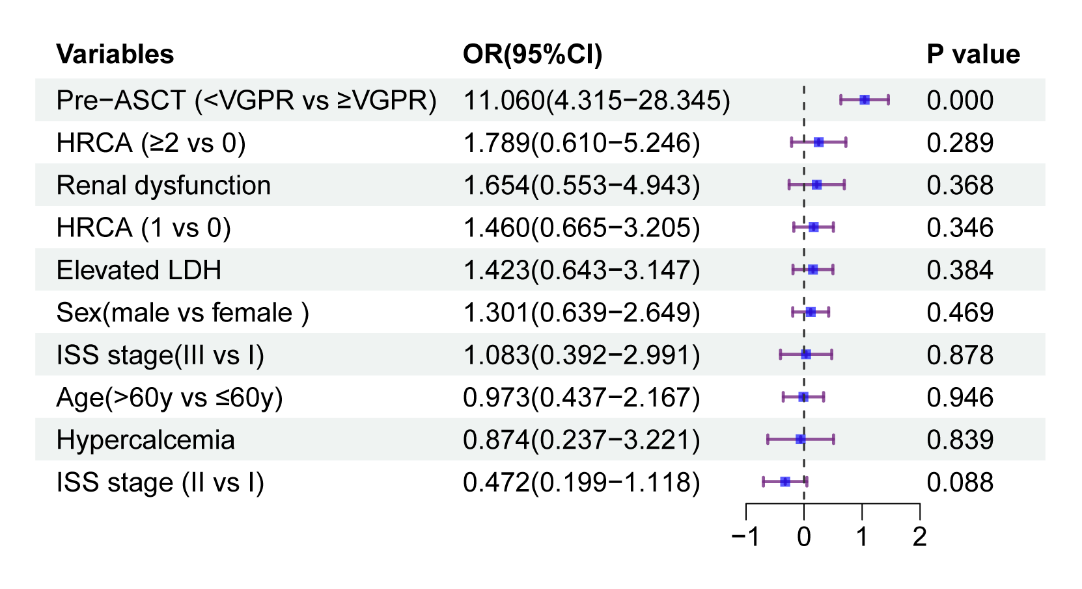


**Fig. S6 Multivariable logistic regression assessing risk factors for MRD positivity post-ASCT.**

OR, odds ratio, OR was transformed into log_10_(OR) in the Figure; CI, confidence interval.
